# Byways of Medical Relief

**Published:** 1911-10-07

**Authors:** 


### BYWAYS OF MEDICAL RELIEF.

### I.—The Little Hospitals.

The voluntary charities of this country have come into being much as the roads have done. A few have been founded on the Roman method, on broad national lines as great highways. But by far the greater number have originated as mere byways, successive feet following each other and beating out a track to lift the fallen, comfort the wretched, and heal the sick. In studying the existing byways of medical relief it becomes at once evident that a continual process of development is at work, turning the byway of to-day into the well-made road of to-morrow. The history of voluntary institutions reveals the small beginnings of many world-famous undertakings, for personal zeal communicates with swift contagion to other men. Thus these lesser manifestations of charity are as springs from which the sources of philanthropy of the future will flow. The little hospital with its half-dozen beds is a great infirmary *in posse*. The humble demonstration of some new form of treatment may result in completely transforming popular ideas.

The byways of relief owe their origin to many different impulses and lead off in a hundred different directions. The rich have the splendid privilege of being able to initiate at will, and have made good use of it in past and present times. To build and support some little institution for relieving suffering which shall be the personal care of the founder is no doubt one of the happiest hobbies open to a man of leisure with brains and money. But the first steps are often marked out by an altruist lacking all but the zeal which can draw others to co-operate with him for some worthy object. Often the impulse is supplied by the dead hand, and the little hospital which has been the day dream of a lifetime rises after the death of the man who has pictured it in every detail. Sometimes it is built as an act of piety to the dead by bereaved survivors. Sometimes it is an act of reasoned forethought, as when a trading company provides hospital accommodation for its employés. Sometimes it is interest in some aspect of treatment which leads a medical man to gather round him a clinic. Sometimes it is a local need, and sometimes a worthy spirit of municipal emulation which sets compassion stirring in a particular direction. It is hard, indeed, to account for the many spontaneous manifestations of altruism which confront us in some localities, as contrasted with the grievous lack in others. From whatever impulse, however, these little undertakings may start, they fall, roughly speaking, under one of two headings. They are either for the relief of persons eligible for ordinary hospital and convalescent relief, or they are intended to supply the necessities of persons who cannot, from the nature of their infirmities, be received in the regular institutions.

Byways of medical relief deal principally with the little institutions founded to pick up the dropped threads in the national schemes for alleviating distress. But it is impossible to ignore the existence of a vast network of little hospitals which fall actually into the category of byways, because they are still in an indeterminate process of formation, and may or may not develop into something of permanent value. The tiny cottage hospital may be considered purely in a tentative stage of existence. It is but a track over the grass and at any moment, if the need for it passes or money cannot be found, it may be closed. These little hospitals are collectively of immense value in extending a chain of relief to remote localities. It has never been possible to compile a complete list of them, for the very fact of their existence may not spread beyond a few miles, but as a sense of local responsibility about health matters develops it may be hoped that each county will shortly take measures to present reports relating to all such institutions within its boundaries. It is of the nature of these little enterprises to be taken up, perpetuated, and improved wherever they meet the real needs of the locality. But this desirable fate does not always overtake them. Curious local jealousies are apt to focus round them, or it may be that the inspiring example of one individual was the sole foundation for the enterprise, and with that removed it may dwindle and perish. Where the latter is the case, where the need was great but means for maintenance have fallen short, there would be a strong case for grants in aid from the county health society, carrying with them inspection and control.

It is hardly surprising, considering the many impulses which lead men to found little hospitals, that there should be some among the number which have failed to justify their existence. Of these we shall have more to say. But it is instructive to notice that the tiny hospital either dies or grows. Good medical and surgical work is only carried on in these little make-shift establishments under such disadvantageous circumstances that doctors refrain from sending cases unless local needs are particularly pressing. Only should such need unmistakably manifest itself will the little hospital become endowed with the spirit of development. Otherwise it remains a somewhat costly failure, and drifts into a kind of adjunct to the workhouse sick wards. In a review of the resources available for use at the hands of the county health society, many derelict institutions of this description will come to light.

The great protection of the little hospitals is the existence in English country society of strong constructive talent, with a big reserve fund of administrative power. True though it may be that little institutions tend to fall on evil times and pass away from the intention of the founders, yet it is equally true that there is ever at work a revivifying influence able to restore, to rebuild, to reorganise wherever things have gone wrong. Outside encouragement, as from a county health society, could evoke in nearly every locality the men and the women to meet the hour. There need be no fear that the future will lack devoted feet to tread down the byways of compassion and strengthen the tracks which the altruistic have marked out for the rescue of sufferers.

